# Comparative Outcomes Following Randomization to a Pilot Facebook-Based HIV Prevention Intervention Among Appalachian Women Involved in the Criminal Legal System

**DOI:** 10.13023/jah.0604.07

**Published:** 2025-01-29

**Authors:** Megan F. Dickson, Erika Pike, Michele Staton

**Affiliations:** University of Kentucky; University of Kentucky; University of Kentucky

**Keywords:** Appalachia, drug use, HIV prevention, re-entry, rural health, social media, women

## Abstract

**Introduction:**

Rural Appalachian women who use drugs and are involved in the criminal legal system are at increased risk for health consequences (such as HIV/Hepatitis C). Service barriers throughout rural communities have prompted a need to examine the effectiveness of novel intervention delivery methods (e.g., social media).

**Purpose:**

This study aims to determine if enhancing an existing HIV prevention intervention with additional modules delivered via Facebook improves service access by examining short-term outcomes among Appalachian women returning to the community following jail release.

**Methods:**

Between 2019 and 2022, consenting women from two rural Appalachian jails were randomly selected, screened, interviewed, and randomized to either the National Institute on Drug Abuse (NIDA) Standard alone (n=30) or the NIDA Standard delivered via Facebook post-incarceration (n=30). Women were included in the final sample after completing both baseline and three-month follow-up interviews (N=50). In 2022, bivariate analyses were used to identify differences in drug use, injection drug use, and drug use before sex across intervention groups, and McNemar’s test was used to measure changes in these risk behaviors within groups over time.

**Results:**

The percent of individuals reporting past three-month HIV-risk behaviors significantly decreased between baseline and follow-up for both groups. There were no between-group differences in risk behaviors.

**Implications:**

Results suggest that high-risk, rural Appalachian women may benefit from HIV prevention interventions delivered via Facebook, particularly during community re-entry following jail release. Facebook intervention delivery is an efficient way to expand the reach of HIV prevention services in a region with known barriers to traditional modes of intervention delivery.

## INTRODUCTION

Rural Appalachia has been disproportionately impacted by the opioid epidemic and is plagued by high rates of polysubstance use, injection drug use (IDU), and overdose.^1^ These substance use risk behaviors have contributed to a significant risk of HIV and Hepatitis C (HCV) transmission throughout the community.^1^ Within rural Appalachia, women are particularly at an increased risk for these negative substance use-related health consequences. This is in part due to their substance use being intertwined with their relationships with risky sexual partners who also oftentimes use substances (e.g., use of injection paraphernalia with a partner^2^–^4^). Women involved in the criminal legal system (CLS) face additional risks, with a recent review pointing to a significantly increased risk of HIV and HCV infection among people who inject drugs who have a history of incarceration.^5^ CLS-involved women also experience unequal access to HIV, HCV, and other health-related services – oftentimes encountering barriers such as stigma, financial instability, and lack of gender-responsive services tailored to their unique needs (e.g., having caregiving responsibilities for children or history of trauma^8^).

In rural communities, the health consequences associated with drug use (e.g., HIV and HCV) are compounded by the barriers to substance use treatment and recovery services that are common to many rural Appalachian communities, including limited transportation options, long travel distances, extensive wait lists, and costs.^7^ During the COVID-19 pandemic, some barriers to health services in rural areas were alleviated through the expansion of telemedicine^8^ while other barriers became even more pronounced. For example, access to the already limited HIV-related services was disrupted, and community syringe access programs and other harm reduction services in rural communities were by and large suspended early in the pandemic.^9^ Post-pandemic, research has shown that challenges in accessing these harm reduction services continue to endure in rural Appalachian communities.^10^,^11^

Due to ongoing challenges in accessing HIV and HCV-related care, there is a need for novel methods of HIV service delivery. Scholars have previously underscored technology as a potential means for HIV service delivery, including social media.^12^ Social media has been used to successfully deliver HIV-risk reduction interventions among other high-risk populations in urban settings,^13^ and Facebook specifically has shown promise for increasing the reach of substance use prevention campaigns.^14^ In rural Appalachia, Facebook has been a successful means of delivering health information^15^ and recruiting and engaging community members in health interventions.^16^ Further, research shows Facebook is commonly used among CLS-involved women in rural Appalachia^17^ and is the preferred social media platform among this population^18^ – thus offering a promising platform for the delivery of a health intervention in this region.^18^,^19^

Despite research signaling the possible utility of Facebook for health intervention delivery and the known barriers to harm reduction services throughout rural Appalachia, there is a limited understanding of how Facebook may be leveraged to expand the reach of HIV prevention interventions among high-risk groups of individuals who may not have access to services in this region (e.g., women who use drugs). The current study aims to fill this gap in the literature by examining short-term outcomes, including risky sexual practices and risky drug use, among rural Appalachian women who participated in a NIDA-funded pilot trial examining an HIV prevention intervention delivered via Facebook. Using an intention-to-treat analysis, outcomes will be examined across groups for behavioral differences at baseline and follow-up. Within-group differences for changes between baseline and follow-up will also be explored.

## METHODS

### Participants and Procedures

**Figure 1:**
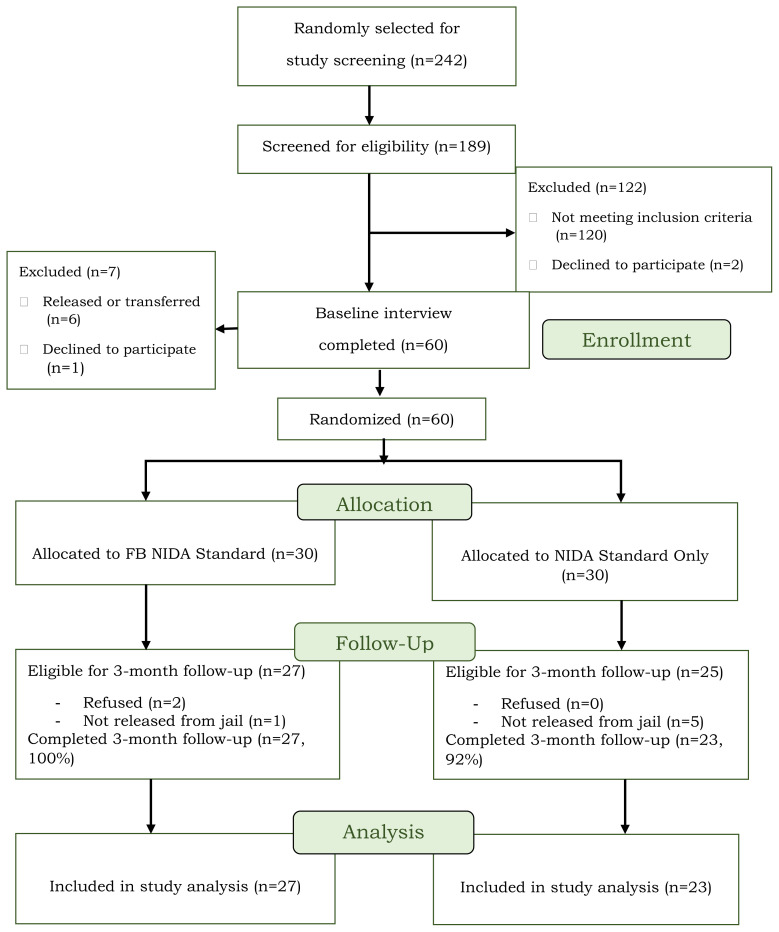
Study CONSORT

Data for this study were collected between June 2019 – April 2022 as part of a NIDA-funded feasibility pilot study examining HIV intervention delivery (NIDA Standard Intervention Model for Injection Drug Users Not in Treatment [NIDA Standard]) via Facebook.^19^ Participants (N=60) were randomly selected from two jails in rural Appalachia and screened for study eligibility. Eligibility criteria included: (1) moderate substance use risk based on the NIDA-modified Alcohol, Smoking and Substance Involvement Screening Test (NM-ASSIST; score of 4+ for any drug^20^); (2) self-reported HIV risk behavior in the past three-months; (3) regular user of Facebook; (4) self-reported HIV negative status; (5) residing in a designated Appalachian county before incarceration; and (6) willingness to participate. Participant enrollment is described in the study CONSORT (Fig. 1 and Staton et al. 2022^18^).

Eligible participants were invited to participate in a face-to-face baseline interview, which was conducted in either a private room at the jail or using Zoom® videoconferencing (following the implementation of COVID-19 restrictions in March 2020). Data were collected by female interviewers from the local Appalachian area using Computer-Assisted Personal Interview (CAPI) software. Research staff, who were certified HIV/HCV counselors, provided HIV/HCV counseling using NIDA Standard content to all participants. Following baseline and counseling, participants were randomized to one of two intervention conditions using Research Randomizer (www.randomizer.org): (1) NIDA Standard alone (n=30), or (2) Facebook (FB) NIDA Standard for 12 weeks post-release from jail (n=30). As described in a previous study published in the *Journal of Clinical and Translational Science*,^19^ participants were informed during the consent process that they would be randomly assigned to an intervention condition, with a 50/50 chance of being assigned to either condition.

Six individuals who enrolled in the pilot were not released to the community, but of those who were released (N=54), all were sent a Facebook friend request from the study team inviting them to the main study page – 30 (55.6%) of whom accepted. While participants in both conditions received an initial intervention session prior to release,^19^ those in the FB NIDA Standard group received up to 12 additional weeks of intervention material (adapted from the NIDA Standard) during community re-entry in a separate, private Facebook intervention group. Participants randomized to the FB NIDA Standard group received the invitation to this private Facebook group following their acceptance of the friend request to the main study page (that individuals in both conditions were able to join), nine of whom accepted. During the first week, these nine participants had access to an introductory video about the intervention and three videos with background information about HIV, Hepatitis B, and HCV, and then for the remainder of the follow-up period, one video per week with adapted content from the NIDA Standard Intervention, tailored to the study population.^18^ Additional information related to intervention content and exposure can be found in Staton et al., 2022.^18^

All participants who were released to the community (N=54) were followed for three months post-jail release, which is consistent with other studies examining the effectiveness of HIV interventions.^3^ Tracking and locating during the followup period was conducted using Facebook,^19^ phone calls, internet and CLS database searches, and flyer mailings. Two individuals who were released to the community refused to participate in the follow-up interview. Of the 52 eligible participants, 50 women completed the follow-up interview (96.0% follow-up rate) and were included in the current analyses.

Participants were paid $25 each for the baseline and follow-up interviews, plus a $25 bonus for completing both interviews. All study screening and data collection procedures were approved by the University of Kentucky Institutional Review Board and protected under a federal Certificate of Confidentiality.

### Measures

#### Demographics

Demographic information was collected at baseline and included age, years of education, sexual orientation (1=identified as heterosexual, 0=identified as another sexuality), relationship status (1=married/living as married, 0=single), employment (1=employed at least part time, 0=unemployed), income, and homelessness (1=homeless, 0=not homeless) during the three months prior to incarceration. Participants were also asked about home computer access (1=yes, 0=no), their primary means of accessing the internet (1=smartphone, 0=other), and number of days they had been incarcerated at the time of their baseline interview. All participants reported being white, so race was not included in analyses.

#### Intervention groups

For this intention-to-treat analysis, participants who completed the follow-up interview were examined by their randomly assigned groups: (1) NIDA Standard alone (n=23) or (2) FB NIDA Standard (n=27).

#### HIV & health risk behaviors

Participants were asked about their substance use and risky sex practices during both the baseline and follow-up interviews. Baseline questions focused on the three months prior to incarceration, and follow-up measures focused on the three months post-release. For the current study, HIV and other health risk measures included: (1) illicit drug use in the past three months (yes/no), (2) injection drug use (IDU) in the past three months (yes/no), and (3) using drugs prior to most recent sexual encounter with either a main or casual partner (yes/no).

### Data Analyses

Two sets of analyses were conducted in 2022 using IBM SPSS Statistics for Windows, Version 28 (IBM Corp., Armonk, NY). For the intention-to-treat analysis, chi-square and *t*-tests were used to compare demographic information and HIV risk behaviors across intervention groups to examine differences between groups at baseline and follow-up. Next, McNemar’s test was used to measure changes in HIV and health risk behaviors between baseline and follow-up within each of the intervention groups. Confidence intervals were also calculated.

## RESULTS

### Sample Characteristics

In Table 1, demographic data are presented by intervention condition for those who completed a follow-up interview (N=50). As shown, the average age of participants was 36, with 12.2 years of education. More than three-quarters identified as heterosexual (76.0%), 18.0% were married, and 6.0% were employed before incarceration. All participants reported being a regular user of Facebook as part of eligibility screening and 86.0% reported that they primarily used a smartphone for internet access. Only education and sexuality differed across groups, with the NIDA Standard only group averaging more years of education (*t*(48)=2.40, *p*=.020) and being more likely to identify as heterosexual (χ^2^(1, N=50)=5.417, *p*=.019). Additional demographic information, such as length of incarceration at baseline interview, can be found in Table 1.

### HIV & Health Risk Behaviors

HIV risk behaviors were common among participants in the three months prior to jail incarceration, with 100.0% reporting illicit drug use, 70.0% reporting IDU, and 92.0% reporting using drugs before their last sexual encounter with either a main or casual partner. During the three-month post-jail release follow-up period, a decreased number of participants reported engaging in HIV risk behaviors overall, with 52.0% reporting drug use, 28.0% reporting IDU, and 32.0% reporting having used drugs prior to their most recent sexual encounter with either a main or casual partner, as shown in Table 1.

#### Between-group differences

The percentage of individuals reporting any drug use during the follow-up period and drug use before their most recent sexual encounter was generally lower among the FB NIDA Standard group compared to the NIDA Standard only group, but differences were not statistically significant.

#### Within-group differences

**Figure 2:**
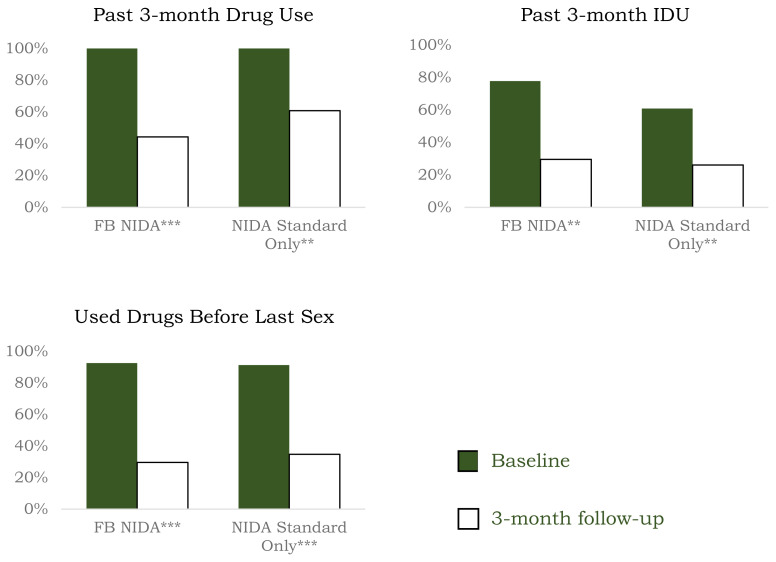
Reductions in Health Risk Behaviors at 3-Month Follow-Up NOTE: ** p < .01; *** p < .001

McNemar’s test indicated there were significant decreases for both intervention groups in the percentage of participants reporting HIV risk behaviors during the follow-up period compared to baseline **(**Fig 2). Specifically, the percentage of individuals reporting past three-month drug use in the NIDA Standard only group decreased 39.1% (*p* = .004) between baseline and follow-up and 55.6% in the FB NIDA Standard group (*p* < .001). Past three-month IDU decreased 57.1% (*p* = .008) among those in the NIDA Standard only group and 67.0% among those in the FB NIDA Standard group (*p* = .002). Finally, there was a 61.9% (*p* < .001) decrease in the percentage of participants in the NIDA Standard only group reporting having used drugs prior to their last sexual encounter and 68.0% (*p* < .001) among those in the FB NIDA Standard group.

## IMPLICATIONS

Widespread substance use risk behaviors and barriers to treatment throughout rural Appalachia have prompted a need for alternative means of delivering harm reduction services. Following the COVID-19 pandemic, a growing body of research has explored social media as an alternative tool for delivering health interventions, and the current study adds to this literature by examining the delivery of an HIV prevention intervention via Facebook. Results suggest that the delivery of an HIV prevention intervention via Facebook is, overall, associated with a similar rate of decreased risk behaviors as face-to-face interventions.^3^

Findings from this feasibility pilot study specifically indicate that the NIDA Standard HIV prevention intervention, when adapted for rural Appalachian women^18^ and delivered via Facebook, is associated with reduced risk behaviors during community re-entry following incarceration among a sample of rural Appalachian women at increased risk of acquiring HIV/HCV. Study results are comparable to those found in an earlier study with a similar population of women, in which the NIDA Standard was delivered face-to-face.^3^ Prior research has pointed to an elevated risk of engaging in HIV risk behaviors during community re-entry^21^ and highlighted concern surrounding a potential HIV outbreak in rural Appalachia.^22^ With evidence of ongoing barriers to harm reduction services throughout the region,^10^ it remains important to identify novel ways to deliver HIV prevention interventions. Successful delivery of such interventions could have broad public health implications in terms of preventing the spread of blood-borne infections such as HIV and HCV.

Although there were within-group statistical differences in risk behaviors between baseline and follow-up, intention-to-treat analyses did not identify significant differences between intervention groups. The ability to detect differences between groups may be limited given that participants in both conditions received a face-to-face session of the NIDA Standard while still incarcerated, and among those randomized to the FB NIDA Standard group, intervention exposure varied, as previously mentioned. Regardless, these results provide additional evidence that the NIDA Standard is a robust intervention and echo earlier research which found that the NIDA Standard was effective at reducing risk behaviors – both alone and when coupled with a motivational interviewing enhancement.^3^ To build upon this study, future research should consider investigating more long-term participant outcomes following HIV prevention interventions delivered via social media, including examining different levels of intervention exposure and whether social media platforms other than Facebook may be more effective for intervention delivery among other high-risk populations in rural Appalachia due to platform preferences (e.g., Instagram vs. Facebook users^23^).

It is also possible that the small sample size of the pilot study limited the ability to discern statistical significance between intervention groups. For example, the percent of individuals reporting past three-month drug use in the FB NIDA Standard group decreased 55.6% between baseline and follow-up compared 39.1% in the NIDA Standard only group. These findings provide further support for the potential of Facebook as an alternative method for delivering a critical service in a community with known barriers to traditional modes of intervention delivery,^24^ particularly given (1) the demonstrated feasibility of using Facebook for intervention delivery,^13^,^19^ (2) evidence of widespread Facebook use in similar populations,^17^,^18^ and (3) the extensive use of smartphones for internet access among study participants. With earlier research concluding that other web-based interventions are effective at reducing HIV risk behaviors among women,^25^ future research should continue to investigate Facebook for intervention delivery using a larger sample size.

### Limitations

There are limitations that should be considered when interpreting study results. First, the generalizability of study results to other CLS-involved women outside the Appalachian Region may be limited due to the purposeful selection of the two jails. Though steps were taken to randomly select women for screening from the jail sites, as described in the previous study published in the *Journal of Clinical and Translational Science*,^19^ it is difficult to ascertain the degree of selection bias due to the limited geographic scope of this study and small sample size of this pilot trial. Second, data were self-reported. While the use of self-reported data related to high-risk drug use and sexual practices is common in this area of research,^2^ it is possible that there may be bias related to social desirability. In addition, concerns related to confidentiality may have impacted participant responses at baseline due to their incarceration. However, participants were assured of IRB protections, as well as the protections of the Certificate of Confidentiality. Baseline questions about the time pre-incarceration may also be subject to recall bias since women had been incarcerated for an average of 109 days at the time of their interview **(**Table 1). Finally, it is also important to note that despite randomization to the FB NIDA Standard group, only a portion of individuals accepted the invitation to the Facebook intervention group and were exposed to the additional content. Given that participant engagement may have been impacted by the COVID-19 pandemic, future research should continue to explore the effectiveness of similar Facebook and other social media-based interventions, including best practices for connecting with incarcerated women post-release to increase their engagement since incarceration may limit the ability to join online social media study pages at enrollment, as has been done in other studies.^13^

## CONCLUSION

Despite limitations, this study demonstrated reductions in overall risk behavior among rural Appalachian women following their participation in this re-entry HIV prevention intervention delivered via Facebook and face-to-face. Even though statistically significant differences did not emerge between the intervention groups, study results are promising for the future of behavioral health services in rural Appalachia. With the limited availability of traditional behavioral health interventions in rural Appalachia, these findings have important implications specifically for HIV/HCV prevention among women throughout the region, suggesting that CLS-involved women who use drugs and other high-risk populations in rural communities may benefit from an HIV intervention delivered via social media. Although future studies should continue to explore the expansion of behavioral health services in rural Appalachia via social media using other platforms and more long-term follow up, results underscore social media as an opportunity to reach rural Appalachian women at risk of acquiring HIV/HCV who are otherwise underserved.

**SUMMARY BOX**

**What is already known about this topic?**

Rural Appalachian women who use drugs are at increased risk of experiencing negative health consequences related to substance use, particularly those involved in the criminal legal system. These risks are further complicated by widespread barriers to substance use prevention, treatment, and health services throughout the region.

**What is added by this report?**

Service barriers have underscored the need for novel intervention delivery methods, including social media platforms. This study specifically contributes to existing literature by exploring short term outcomes (e.g., risky drug use and risky sexual practices) among a sample of women who participated in a NIDA-funded pilot trial examining an HIV prevention intervention delivered via Facebook upon their return to the community following release from jail.

**What are the implications for future research?**

Findings suggest that delivering an HIV prevention intervention via Facebook is a promising alternative for traditional behavioral health interventions in rural Appalachia, as it was successful at reducing HIV and other health risk behaviors. This underscores social media as an opportunity to reach high-risk populations in rural communities who are otherwise underserved.

## Figures and Tables

**Table 1:** Demographic and Outcome Information (N=50)

	FB NIDA (n=27)	NIDA Only (n=23)	Total (n=50)		
	*% / M (SD)*	*% / M (SD)*	*% / M (SD)*	*p*	95% CI
**Demographics** *(baseline)*					
Age	33.9 (7.4)	38.2 (7.7)	35.9 (7.8)	.054	−.08 – 8.57
**Education (years)**	**11.6 (2.0)**	**12.9 (1.8)**	**12.2 (2.0)**	**.020**	**.21** – **2.36**
**% heterosexual**	**63.0%**	**91.3%**	**76.0%**	**.019**	**.03** –**.84**
% married	18.5%	17.4%	18.0%	.918	.25 – 4.61
% employed at least part time (3 months before incarceration)	7.4%	4.3%	6.0%	.650	.15 – 20.76
Income (3 months before incarceration) incarceration)	$9,654 ($19,260)	$3,572 ($4,674)	$6,856 ($14,697)	.123	−14,368.05 – 2,204.43
% homeless or living with someone to avoid being homeless (3 months before	55.6%	30.4%	44.0%	.075	.75 – 7.37
% had computer at home	51.9%	65.2%	58.0%	.340	.18 – 1.80
% primarily use phone to access internet	85.2%	87.0%	86.0%	.857	.17 – 4.33
Days incarcerated at baseline (average)	108.7 (156.7)	109.3 (148.4)	108.9 (151.2)		−87.40 – 88.61
**Primary Outcomes** *(baseline)*					
% past 3-month drug use	100.0%	100.0%	100.0%	-	-
% past 3-month IDU	77.8%	60.9%	70.0%	.193	.66 – 7.73
% used drugs before last sexual encounter	92.6%	91.3%	92.0%	.867	.15 – 9.19
**Primary Outcomes** *(follow-up)*					
% past 3-month drug use	44.4%	60.9%	52.0%	.247	.17 – 1.59
% past 3-month IDU	29.6%	26.1%	28.0%	.781	.34 – 4.14
% used drugs before last sexual encounter	29.6%	34.8%	32.0%	.697	.24 – 2.60
